# A Case of simultaneous occurrence of Marine – Lenhart syndrome and a papillary thyroid microcarcinoma

**DOI:** 10.1186/1472-6823-13-16

**Published:** 2013-05-08

**Authors:** Thomas Scherer, Evelyne Wohlschlaeger-Krenn, Michaela Bayerle-Eder, Christian Passler, Angelika Reiner-Concin, Michael Krebs, Alois Gessl

**Affiliations:** 1 Division of Endocrinology and Metabolism, Department of Internal Medicine III, Medical University of Vienna, Waehringer Guertel 18-20, Vienna, 1090, Austria; 2 Department of Surgery, SMZ Floridsdorf, Hinaysgasse 1, Vienna, 1210, Austria; 3 Institute of Pathology, Danube Hospital, Langobardenstr. 122, Vienna, 1220, Austria

**Keywords:** Toxic adenoma, Graves’ disease, Marine-Lenhart syndrome, Papillary carcinoma, Hyperthyroidism

## Abstract

**Background:**

Marine-Lenhart syndrome is defined as the co-occurrence of Graves’ disease and functional nodules. The vast majority of autonomous adenomas are benign, whereas functional thyroid carcinomas are considered to be rare. Here, we describe a case of simultaneous occurrence of Marine-Lenhart syndrome and a papillary microcarcinoma embedded in a functional nodule.

**Case presentation:**

A 55 year-old, caucasian man presented with overt hyperthyroidism (thyrotropin (TSH) <0.01 μIU/L; free thyroxine (FT4) 3.03 ng/dL), negative thyroid peroxidase and thyroglobulin autoantibodies, but elevated thyroid stimulating hormone receptor antibodies (TSH-RAb 2.6 IU/L). Ultrasound showed a highly vascularized hypoechoic nodule (1.1 × 0.9 × 2 cm) in the right lobe, which projected onto a hot area detected in the ^99m^technetium thyroid nuclear scan. Overall uptake was increased (4.29%), while the left lobe showed lower tracer uptake with no visible background-activity, supporting the notion that both Graves’ disease and a toxic adenoma were present. After normal thyroid function was reinstalled with methimazole, the patient underwent thyroidectomy. Histological work up revealed a unifocal papillary microcarcinoma (9 mm, pT1a, R0), positively tested for the BRAF V600E mutation, embedded into the hyperfunctional nodular goiter.

**Conclusions:**

Neither the finding of an autonomously functioning thyroid nodule nor the presence of Graves’ disease rule out papillary thyroid carcinoma.

## Background

The eponym Marine-Lenhart syndrome is typically used for the rare condition of concurrent manifestation of Graves’ disease and thyroid autonomy (Plummer’s disease) [1-3]. In general, the majority of autonomic adenomas of the thyroid are benign, whereas autonomic thyroid carcinomas are considered a relatively rare finding [4,5]. Here, we report the exceptional case of simultaneous occurrence of Marine-Lenhart syndrome and a papillary microcarcinoma, which was embedded in an autonomous adenoma.

## Case presentation

**Figure 1:**
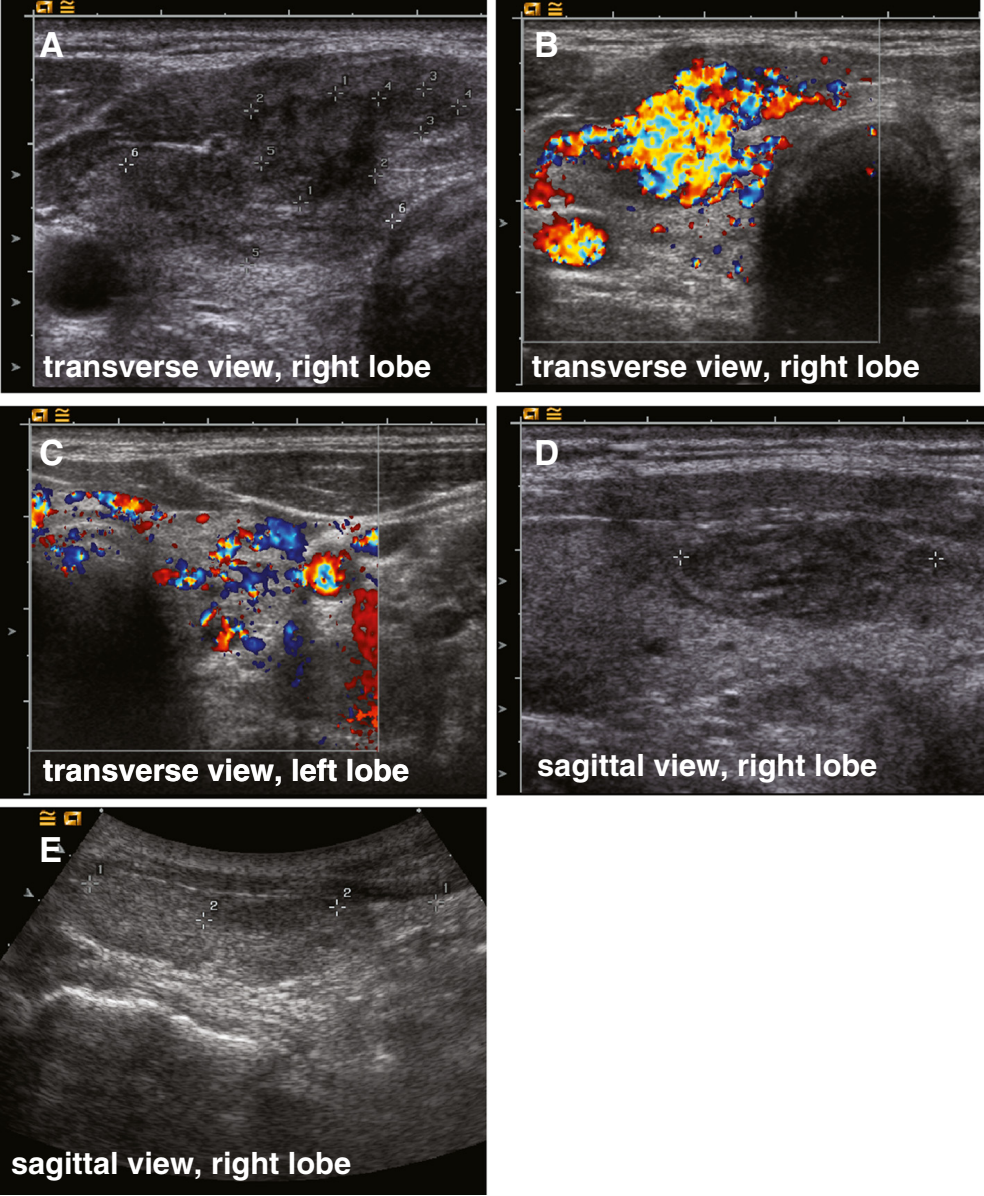
**Ultrasound.** Thyroid ultrasound transverse (**A**, **B** and **C**) and sagittal view (**D** and **E**) of the right and left thyroid lobe showing a diffusely heterogenous gland with a clearly demarcated hypoechoic hypervascularized nodule in an anterior caudal position of the right lobe.

**Figure 2:**
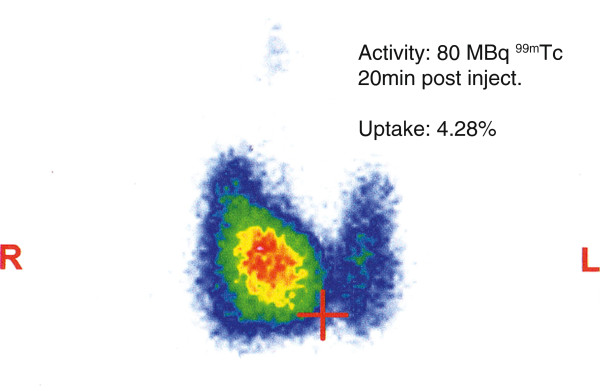
**Thyroid Scan.** Thyroid nuclear scan, showed an orthotopic, V-shaped thyroid gland with accentuated radiotracer uptake into the right lobe. The uptake within the right lobe was heterogeneous with a focal area of relatively increased tracer accumulation centro-caudally, which projected onto the location of the hypervascularized adenoma, detected in the ultrasound. Background-activitiy was completely missing, which is compatible with Graves’ disease. A small pyramidal lobe is emanating from the right lobe.

A 55-year-old Caucasian man was referred because of abnormal thyroid function tests and intermittent atrial fibrillation. He reported of occasional palpitations, episodes of tachycardia (up to 200 bpm), especially under exertion, and excessive sweating. The patient was previously started on bisoprolol 2.5 mg once daily and presented with normofrequent sinus rhythm. On physical examination his thyroid was moderately enlarged (WHO grade Ib) and soft. Thyroid function tests revealed hyperthyroidism with complete suppression of thyroid stimulating hormone (TSH) (<0.01 μIU/mL, see Table 1 for reference range), elevated FT4 (3.03 ng/dL) and increased total thyroxine (TT4) and total triiodothyronine (TT3) levels (132 and 2.53 ng/mL respectively). Thyroid peroxidase (TPO) and thyroglobulin autoantibodies (TgAb) were negative, but TSH-RAbs were elevated (2.6 IU/L), indicating that the patient suffered from Graves’ disease (See Table 1). Upon initial admission the patient showed no clinical signs of Graves’ orbitopathy. A thyroid ultrasound showed multiple hypoechoic nodules in both thyroid lobes. The most prominent nodule appeared in anterior caudal position of the right lobe embedded in a diffusely heterogeneous thyroid gland. The lesion measured 1.1 × 0.9 × 2 cm, showed no signs of microcalcification, but was highly vascularized in the Doppler color flow, whereas the rest of the thyroid tissue showed moderate signs of hypervascularization (Figure 1). A ^99m^Technetium thyroid nuclear scan displayed an orthotopic, V-shaped thyroid with asymmetrical uptake and a hot area in the right lower lobe compatible with the hypoechoic, hypervascularized lesion discovered in ultrasonography (Figure 2). Uptake in the remaining thyroid tissue was comparatively lower but not suppressed, with increased overall uptake (4.29%, reference range: 0.5% – 2%) and almost no detectable background activity. Thus, both signs of Graves’ disease and thyroid autonomy were coexisting in our patient hence the diagnosis Marine-Lenhart syndrome.

**Table 1:** Laboratory values

**Timepoint**	**Intial diagnosis**	**Follow up 1 (after 3 weeks)**	**Follow up 2 (after 9 weeks)**	**Post surgical (after 17 weeks)**
FT4 (0.76 – 1.66 ng/dL)	3.03	2.09	1.44	1.27
TSH (0.44 – 3.77 μIU/mL)	<0.01	<0.01	<0.01	7.26
TT4 (58 – 124 ng/mL)	132	109	90	80
TT3 (0.8 – 1.8 ng/mL)	2.53	1.91	1.49	0.89
TgAb (< 33 IU/mL)	<10	ND	ND	<10
TPO (< 28 IU/mL)	9	ND	ND	ND
TSH-RAb (< 1.75 IU/L)	2.6	ND	ND	ND
TBG (14 – 32 μg/mL)	19.7	19	ND	28.7
Tg (1.6 – 55 ng/mL)	ND	ND	ND	5.7
Calcitonin (< 8 pg/mL)	2.7	ND	ND	ND

*ND – not determined*.

**Figure 3:**
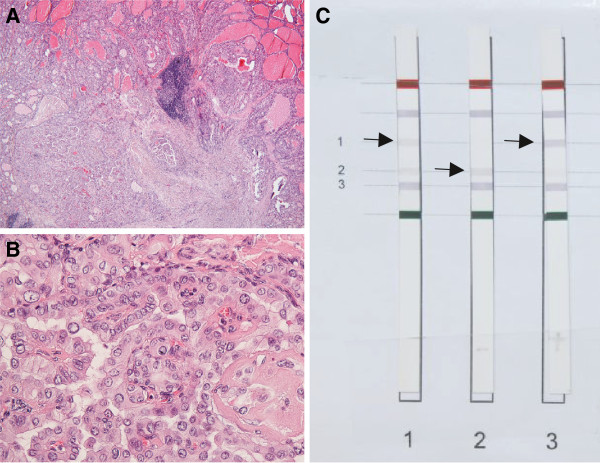
**Histology. (A, B)** H&E histology of papillary thyroid carcinoma, **A**, characteristic morphology at low power, **B** Nuclear morphology at high power. (**C)** Strip Assay demonstrating BRAF V600E mutation: Strip #**1** patient, strip #**2** negative control, strip #**3** positive control; Formalin fixed paraffin embedded sections of carcinoma tissue were evaluated for the BRAF V600E mutation by using the ViennaLab BRAF Strip Assay.

The patient was then started on 20 mg methimazole twice daily for the first seven days after his initial presentation, followed by 20 mg methimazole once daily for the following 8 weeks, which completely resolved his hyperthyroid condition until follow up 2 (Table 1). However, because of the unlikeliness of a permanent remission, the existence of multiple “cold” nodules and the relative resistance of patients with coexisting Graves’ disease and toxic adenoma to radioiodine therapy [2], a definitive, surgical treatment was aspired. The patient therefore underwent thyroidectomy three months after initial diagnosis. The patient received alternating 10 mg and 20 mg methimazole once daily until the thyroid gland was removed. After thyroid surgery bisoprolol 2.5 mg once daily was stopped. Notably, embedded in the nodular area of the right thyroid lobe a, macroscopically, white and firm elastic area was discovered, which was diagnosed as a papillary microcarcinoma (5 mm) in an intraoperative frozen section. Histological work-up revealed a unifocal papillary microcarcinoma (9 mm, pT1a, R0) in the right thyroid lobe with signs of a hyperfunctioning nodular goiter (see Figure 3 A and B). Furthermore, the carcinoma sections were positively tested for the BRAF V600E mutation using the ViennaLab BRAF Strip Assay (Vienna, Austria) (Figure 3C). After a short postoperative recovery phase the patient was discharged under levothyroxine therapy.

A postoperative follow up 17 weeks after the initial diagnosis showed subclinical hypothyroidism, which was fixed by adjusting the levothyroxine therapy (Table 1). Notably, the patient developed a moderately active orbitopathy postoperatively, which was treated with IV corticosteroids for 3 months.

## Discussion

The BRAF V600E mutation is usually associated with higher malignancy and aggressiveness in papillary thyroid carcinomas [6] and in most cases with typical ultrasound features of thyroid malignancy [7]. However, due to the unifocality of the lesion and the fact that the papillary carcinoma in our patient measured less than 10 mm, according to the 2009 consensus of the American Thyroid Association no radioiodine therapy was performed [8]. Therefore, postsurgical thyroglobulin (TG) levels are still detectable in our patient (Table 1) and only of limited use in postsurgical follow up.

It is commonly believed that papillary microcarcinomas only rarely collocate within toxic thyroid adenomas, although several case reports are found in the literature [9-16]. A selection of retrospective studies estimate the risk of concurrent papillary carcinomas in functioning nodules ranging from 0.34% up to 5% [5,17,18]. In a cohort of US children with toxic adenoma the malignancy rate was yet higher (about 11%) [19]. Thus, despite the relatively low risk of malignant transformation of functioning nodules, these lesions need careful clinical follow up with ultrasound and possibly fine needle aspiration biopsy especially when patients are young.

Furthermore, it is still subject of debate whether the frequency of thyroid cancer is higher in patients with Graves’ disease, mostly because the studies are typically retrospective and extend over a long period of time, where diagnostic capabilities and tools might have changed significantly (extensively reviewed in [20]). However, it seems that the prevalence of palpable nodules is about 3-fold higher in Graves’ patients (15.8%) [21] compared to the general population living in a non-endemic region (5%) [22]. Studies relying on ultrasound imaging also find a higher prevalence for thyroid nodules in patients suffering form Graves’ disease (33.6%) [23] compared to the general population (about 26%) although to a lesser extent [24,25]. Of note, in the former study more than half the patients suffering from Graves’ disease developed thyroid nodules during the 2-year follow up, which suggests that Graves’ disease promotes *de novo* thyroid nodule formation [23]. Furthermore, the mean malignancy rate of palpable nodules in Graves’ patients is about 16% versus a 5% malignancy rate in the general population (reviewed in [26]). Thus, in conjunction with the above-mentioned prevalence rates of thyroid nodules, it can be estimated that Graves’ patients have an almost 10-fold higher chance of developing thyroid cancer. In a retrospective multicenter study with more than 500 Graves’ patients thyroid carcinomas were found in 3.8% (20 out of 21 were papillary carcinomas) [27]. Another study matched these results and found a malignancy rate of 3.3% in their collective of Graves’ patients [28], whereas the incidence of thyroid cancer in the general US population was estimated at about 14.4 per 100,000 [29]. Of note, in the former study all papillary carcinomas were detected within thyroid nodules embedded into a Graves’ goiter. In total 15% of the patients suffering from both, nodules and Graves’ disease, also had thyroid carcinomas [27]. In another study the risk for malignancy of a thyroid nodule within a toxic diffuse goiter even reached 22% [30], therefore any nodule within a Graves’ goiter should be carefully examined to rule out a thyroid carcinoma.

There is evidence that thyroid cancer in Graves’ patients is more aggressive. Therefore, an important question is, whether the presence of Graves’ disease in thyroid cancer patients affects their clinical outcome. Studies have found that in Graves’ patients thyroid carcinomas grow more invasively and develop lymph node and distant metastases more frequently compared to euthyroid controls [31-33], although a more recent study was not able to confirm these data [34]. Yet, given the possibility that Graves’ disease increases nodule formation and their respective risk for malignant transformation, it is conceivable that Graves’ disease may constitute an additional risk factor when managing thyroid cancer patients lowering the threshold for surgical intervention.

Color flow Doppler sonography has proven a useful tool in the differential diagnosis of toxic multinodular goiter. Boi et al. have indicated that hyperthyroid patients with multinodular goiter can be subdivided into two distinct groups using color flow Doppler patterns: 1) Nodules with normal vascularization surrounded by hypervascularized hypoechoic thyroid tissue showing a similar picture as in Graves’ patients. Notably, in 44% of these patients TSH receptor antibodies were elevated; and 2) heterogenous hypervascularized nodules embedded into normoechoic-normovascular thyroid parenchyma. None of these patients were tested positive for TSH receptor antibodies [35]. The first group constitutes a group of patients, who probably developed Graves’ disease in a non-toxic multinodular goiter. Our patient on the other hand showed features of both groups. His prominent nodule was heterogeneous and clearly more vascularized compared to the surrounding thyroid parenchyma matching the ultrasound criteria of group 2). However, his TSH receptor antibody titers were also elevated, indicating that both thyroid autonomy and Graves’ disease are concurrent.

Finally, hyperthyroid patients have a disproportionate increase in T3 compared to T4 levels [36]. Laurberg et al. have found that this probably results from a relative increase in type 1 iodothyronine deiodinase activity in hyperthyroid patients compared to healthy subjects, where type 2 iodothyronine deiodinase accounts for the majority of T3 production [37,38]. Our patient with Marine-Lenhart syndrome also presented with an increased T3 to T4 ratio of 1.9%. This matches what Laurberg et al. have found in their collective of multinodular toxic goiter. Their patients with thyroid autonomy had a lower T3/T4 ratio compared to those with Graves’ disease (2.0 versus 2.7%) [38], suggesting that in our patient the autonomous adenoma was the more prominent component of disease, which is also supported by the relatively low levels of circulating TSH-RAb and thyroid uptake in the region outside of the adenoma.

## Conclusion

Taken together, neither the finding of an autonomously functioning thyroid nodule nor the presence of Graves’ disease can rule out a papillary thyroid carcinoma. Although, there is still some debate whether Graves’ disease increases the risk for thyroid cancer, nodules embedded in a Graves’ goiter should be carefully evaluated for malignant transformation especially when patients are young.

### Consent

Written informed consent was obtained from the patient for publication of this Case report and any accompanying images. A copy of the written consent is available for review by the Series Editor of this journal.

## Abbreviations

FT4: Free thyroxine; TSH: Thyrotropin or thyroid-stimulating hormone; TT4: Total thyroxine; TT3: Total triiodothyronine; TgAb: Thyroglobulin antibody; TPO: Thyroperoxidase antibody; TSH-RAb: Thyroid stimulating hormone receptor antibody; TBG: Thyroxine-binding globulin; Tg: Thyroglobulin; ND: Not determined.

## Competing interest

The authors declare that no financial conflict of interest exists.

## Authors’ contributions

T.S. compiled data and wrote the manuscript, E.W.K. and M.B.E. researched data and contributed to discussion, C.P. performed thyroid surgery, A.R.C. performed the histologic and molecular analyses, M.K. edited the manuscript and contributed to discussion, A.G. identified the case and wrote the manuscript. All authors read and approved the final manuscript.

## Pre-publication history

The pre-publication history for this paper can be accessed here:

http://www.biomedcentral.com/1472-6823/13/16/prepub

## References

[B1] Biersack HJ Biermann K The Marine-Lenhart syndrome revisited Wien Klin Wochenschr 2011 123 459 462 10.1007/s00508-011-0029-5 21766231

[B2] Charkes ND Graves’ disease with functioning nodules (Marine-Lenhart syndrome) J Nucl Med 1972 13 885 892 4678244

[B3] Marine D Lenhart CH Pathological anatomy of exophthalmic goiter: the anatomical and physiological relations of the thyroid gland to the disease; the treatment Arch Intern Med 1911 VIII 265 316 10.1001/archinte.1911.00060090002001

[B4] Harach HR Sanchez SS Williams ED Pathology of the autonomously functioning (hot) thyroid nodule Ann Diagn Pathol 2002 6 10 19 10.1053/adpa.2002.30605 11842375

[B5] Erdogan MF Anil C Ozer D Kamel N Erdogan G Is it useful to routinely biopsy hot nodules in iodine deficient areas? J Endocrinol Invest 2003 26 128 131 12739739 10.1007/BF03345140

[B6] Frasca F Nucera C Pellegriti G Gangemi P Attard M Stella M Loda M Vella V Giordano C Trimarchi F Mazzon E Belfiore A Vigneri R BRAF(V600E) mutation and the biology of papillary thyroid cancer Endocr Relat Cancer 2008 15 191 205 10.1677/ERC-07-0212 18310287

[B7] Kabaker AS Tublin ME Nikiforov YE Armstrong MJ Hodak SP Stang MT McCoy KL Carty SE Yip L Suspicious Ultrasound Characteristics Predict BRAF V600E-Positive Papillary Thyroid Carcinoma Thyroid 2012 22 6 585 589 Epub 2012 Apr 23 10.1089/thy.2011.0274. 22524468 PMC3358112

[B8] Cooper DS Doherty GM Haugen BR Kloos RT Lee SL Mandel SJ Mazzaferri EL McIver B Pacini F Schlumberger M Sherman SI Steward DL Tuttle RM Revised american thyroid association management guidelines for patients with thyroid nodules and differentiated thyroid cancer Thyroid 2009 19 1167 1214 10.1089/thy.2009.0110 19860577

[B9] De Rosa G Testa A Maurizi M Satta MA Aimoni C Artuso A Silvestri E Rufini V Troncone L Thyroid carcinoma mimicking a toxic adenoma Eur J Nucl Med 1990 17 179 184 10.1007/BF00811447 2279499

[B10] Kim TS Asato R Akamizu T Harada D Nakashima Y Higashi T Yamamoto N Tamura Y Tamaki H Hirano S Tanaka S Ito J A rare case of hyperfunctioning papillary carcinoma of the thyroid gland Acta Otolaryngol Suppl 2007 557 55 57 17453446 10.1080/03655230601066785

[B11] Lamberg BA Makinen J Murtomaa M Papillary thyroid carcinoma in a toxic adenoma Nuklearmedizin 1976 15 138 141 958901

[B12] Laroche C Cremer GA Sereni D Dero M Papillary thyroid cancer lying within a toxic adenoma (author’s transl) Ann Med Interne (Paris) 1979 130 675 678 539686

[B13] Majima T Doi K Komatsu Y Itoh H Fukao A Shigemoto M Takagi C Corners J Mizuta N Kato R Nakao K Papillary thyroid carcinoma without metastases manifesting as an autonomously functioning thyroid nodule Endocr J 2005 52 309 316 10.1507/endocrj.52.309 16006725

[B14] Nishida AT Hirano S Asato R Tanaka S Kitani Y Honda N Fujiki N Miyata K Fukushima H Ito J Multifocal hyperfunctioning thyroid carcinoma without metastases Auris Nasus Larynx 2008 35 432 436 10.1016/j.anl.2007.07.008 17826928

[B15] Tfayli HM Teot LA Indyk JA Witchel SF Papillary thyroid carcinoma in an autonomous hyperfunctioning thyroid nodule: case report and review of the literature Thyroid 2010 20 1029 1032 10.1089/thy.2010.0144 20718686 PMC2933378

[B16] Uludag M Yetkin G Citgez B Isgor A Basak T Autonomously functioning thyroid nodule treated with radioactive iodine and later diagnosed as papillary thyroid cancer Hormones (Athens) 2008 7 175 179 18477556 10.1007/BF03401510

[B17] Rosler H Wimpfheimer C Ruchti C Kinser J Teuscher J Hyperthyroidism in thyroid cancer. Retrospective study of 53 cases Nuklearmedizin 1984 23 293 300 6549407

[B18] Mizukami Y Michigishi T Nonomura A Yokoyama K Noguchi M Hashimoto T Nakamura S Ishizaki T Autonomously functioning (hot) nodule of the thyroid gland. A clinical and histopathologic study of 17 cases Am J Clin Pathol 1994 101 29 35 8279452 10.1093/ajcp/101.1.29

[B19] Croom RD 3rd Thomas CG Jr Reddick RL Tawil MT Autonomously functioning thyroid nodules in childhood and adolescence Surgery 1987 102 1101 1108 3686350

[B20] Pazaitou-Panayiotou K Michalakis K Paschke R Thyroid cancer in patients with hyperthyroidism Horm Metab Res 2012 44 255 262 22334393 10.1055/s-0031-1299741

[B21] Dobyns BM Sheline GE Workman JB Tompkins EA McConahey WM Becker DV Malignant and benign neoplasms of the thyroid in patients treated for hyperthyroidism: a report of the cooperative thyrotoxicosis therapy follow-up study J Clin Endocrinol Metab 1974 38 976 998 10.1210/jcem-38-6-976 4134013

[B22] Vander JB Gaston EA Dawber TR The significance of nontoxic thyroid nodules. Final report of a 15-year study of the incidence of thyroid malignancy Ann Intern Med 1968 69 537 540 10.7326/0003-4819-69-3-537 5673172

[B23] Cantalamessa L Baldini M Orsatti A Meroni L Amodei V Castagnone D Thyroid nodules in Graves disease and the risk of thyroid carcinoma Arch Intern Med 1999 159 1705 1708 10.1001/archinte.159.15.1705 10448772

[B24] Brander A Viikinkoski P Nickels J Kivisaari L Thyroid gland: US screening in a random adult population Radiology 1991 181 683 687 1947082 10.1148/radiology.181.3.1947082

[B25] Chung WY Chang HS Kim EK Park CS Ultrasonographic mass screening for thyroid carcinoma: a study in women scheduled to undergo a breast examination Surg Today 2001 31 763 767 10.1007/s005950170044 11686552

[B26] Belfiore A Russo D Vigneri R Filetti S Graves’ disease, thyroid nodules and thyroid cancer Clin Endocrinol (Oxf) 2001 55 711 718 10.1046/j.1365-2265.2001.01415.x 11895209

[B27] Kraimps JL Bouin-Pineau MH Mathonnet M De Calan L Ronceray J Visset J Marechaud R Barbier J Multicentre study of thyroid nodules in patients with Graves’ disease Br J Surg 2000 87 1111 1113 10.1046/j.1365-2168.2000.01504.x 10931060

[B28] Kim WB Han SM Kim TY Nam-Goong IS Gong G Lee HK Hong SJ Shong YK Ultrasonographic screening for detection of thyroid cancer in patients with Graves’ disease Clin Endocrinol (Oxf) 2004 60 719 725 10.1111/j.1365-2265.2004.02043.x 15163336

[B29] Jemal A Siegel R Xu J Ward E Cancer statistics, 2010 CA Cancer J Clin 2010 60 277 300 10.3322/caac.20073 20610543

[B30] Pacini F Elisei R Di Coscio GC Anelli S Macchia E Concetti R Miccoli P Arganini M Pinchera A Thyroid carcinoma in thyrotoxic patients treated by surgery J Endocrinol Invest 1988 11 107 112 3361079 10.1007/BF03350115

[B31] Ozaki O Ito K Kobayashi K Toshima K Iwasaki H Yashiro T Thyroid carcinoma in Graves’ disease World J Surg 1990 14 437 440 discussion 440–431 10.1007/BF01658550 2368449

[B32] Pellegriti G Belfiore A Giuffrida D Lupo L Vigneri R Outcome of differentiated thyroid cancer in Graves’ patients J Clin Endocrinol Metab 1998 83 2805 2809 10.1210/jc.83.8.2805 9709951

[B33] Belfiore A Garofalo MR Giuffrida D Runello F Filetti S Fiumara A Ippolito O Vigneri R Increased aggressiveness of thyroid cancer in patients with Graves’ disease J Clin Endocrinol Metab 1990 70 830 835 10.1210/jcem-70-4-830 2180978

[B34] Yano Y Shibuya H Kitagawa W Nagahama M Sugino K Ito K Recent outcome of Graves’ disease patients with papillary thyroid cancer Eur J Endocrinol 2007 157 325 329 10.1530/EJE-07-0136 17766715

[B35] Boi F Loy M Piga M Serra A Atzeni F Mariotti S The usefulness of conventional and echo colour Doppler sonography in the differential diagnosis of toxic multinodular goitres Eur J Endocrinol 2000 143 339 346 10.1530/eje.0.1430339 11022175

[B36] Nicoloff JT Low JC Dussault JH Fisher DA Simultaneous measurement of thyroxine and triiodothyronine peripheral turnover kinetics in man J Clin Invest 1972 51 473 483 10.1172/JCI106835 4110897 PMC302152

[B37] Maia AL Kim BW Huang SA Harney JW Larsen PR Type 2 iodothyronine deiodinase is the major source of plasma T3 in euthyroid humans J Clin Invest 2005 115 2524 2533 10.1172/JCI25083 16127464 PMC1190373

[B38] Laurberg P Vestergaard H Nielsen S Christensen SE Seefeldt T Helleberg K Pedersen KM Sources of circulating 3,5,3′-triiodothyronine in hyperthyroidism estimated after blocking of type 1 and type 2 iodothyronine deiodinases J Clin Endocrinol Metab 2007 92 2149 2156 10.1210/jc.2007-0178 17389703

